# Evaluating the Performance of Rice Genotypes for Improving Yield and Adaptability Under Direct Seeded Aerobic Cultivation Conditions

**DOI:** 10.3389/fpls.2019.00159

**Published:** 2019-02-15

**Authors:** Nitika Sandhu, Ram Baran Yadaw, Bedanand Chaudhary, Hari Prasai, Khandakar Iftekharuddaula, Challa Venkateshwarlu, Anandan Annamalai, Phetmanyseng Xangsayasane, Khushi Ram Battan, Mangat Ram, Ma. Teresa Sta. Cruz, Paquito Pablico, Paul Cornelio Maturan, K. Anitha Raman, Margaret Catolos, Arvind Kumar

**Affiliations:** ^1^Rice Breeding Platform, International Rice Research Institute, Los Banos, Philippines; ^2^National Rice Research Program, Hardinath, Nepal; ^3^Regional Agriculture Research Station, Tarahara, Nepal; ^4^Plant Breeding Division, Bangladesh Rice Research Institute, Gazipur, Bangladesh; ^5^South Asia Breeding Hub, International Rice Research Institute, ICRISAT, Hyderabad, India; ^6^Crop Improvement Division, National Rice Research Institute, Cuttack, India; ^7^Agriculture Research Center, National Agriculture and Forestry Research Institute, Vientiane, Laos; ^8^Rice Research Station, Kaul, India

**Keywords:** aerobic, gene, grain yield, QTLs, rice, stability, water scarcity

## Abstract

With the changing climatic conditions and reducing labor-water availability, the potential contribution of aerobic rice varieties and cultivation system to develop a sustainable rice based agri-food system has never been more important than today. Keeping in mind the goal of identifying high-yielding aerobic rice varieties for wider adaptation, a set of aerobic rice breeding lines were developed and evaluated for grain yield, plant height, and days to 50% flowering in 23 experiments conducted across different location in Philippines, India, Bangladesh, Nepal, and Lao-PDR between 2014 and 2017 in both wet and dry seasons. The heritability for grain yield ranged from 0.52 to 0.90. The season-wise two-stage analysis indicated significant genotype x location interaction for yield under aerobic conditions in both wet and dry seasons. The genotype × season × location interaction for yield was non-significant in both seasons indicating that across seasons the genotypes at each location did not show variability in the grain yield performance. Mean grain yield of the studied genotypes across different locations/seasons ranged from 2,085 to 6,433 Kg ha^−1^. The best-fit model for yield stability with low AIC value (542.6) was AMMI(1) model. The identified stable genotypes; IR 92521-143-2-2-1, IR 97048-10-1-1-3, IR 91326-7-13-1-1, IR 91326-20-2-1-4, and IR 91328-43-6-2-1 may serve as novel breeding material for varietal development under aerobic system of rice cultivation. High yield and stable performance of promising breeding lines may be due to presence of the earlier identified QTLs including grain yield under drought, grain yield under aerobic conditions, nutrient uptake, anaerobic germination, adaptability under direct seeded conditions, and tolerance to biotic stress resistance such as *qDTY*_2.1_*, qDTY*_3.1_*, qDTY*_12.1_*, qNR*_5.1_*, AG*_9.1_*, qEVV*_9.1_*, qRHD*_1.1_*, qRHD*_5.1_, *qRHD*_8.1_ *qEMM*_1.1_*, qGY*_6.1_*, BPH3, BPH17, GM4, xa4, Xa21, Pita, and Pita2*. The frequency of *xa4* gene was highest followed by *qAG*_9.1_, *GM4, qDTY*_3.1_*, qDTY*_2.1_*, qGY*_6.1_, and *qDTY*_12.1_.

## Introduction

Global climate change, water scarcity, labor shortage, declining water-table level, predicted significant shortfall in rice production, increasing population, expensive labor, and increasing green-house gas emission (GHG; Monaco et al., 2016) are some of the possible reasons focusing the introduction of water-labor-energy efficient direct seeded aerobic rice cultivation system (Nie et al., 2012; Rasul, 2016; USGS, 2016). By 2050, the worldwide water requirement is expected to increase by 55% (Connor, 2015; WWAP, 2016). By 2050, there is a strong necessity to boost the food production by 60% globally and 100% in the developing countries of the world (Alexandratos and Bruinsma, 2012).

In addition to water scarcity, declining agriculture involved labor forces in Asia by 0.1–0.4% (0.2% per year); in Bangladesh, Thailand, and Malaysia by 0.25–0.40%; in Cambodia and Philippines by 0.18% together with the escalating labor wages are important concerns for the sustainability of puddled transplanted rice (PTR) cultivation system (Kumar and Ladha, 2011). Traditional puddled system of rice cultivation requires 114-man days/ha as against 68-man days/ha in direct seeded aerobic cultivation system (Yamano et al., 2013).

Agricultural productivity is a result of combined efforts involving improvement of water use efficiency, land and water management practices, exploitation of water saving technologies, introduction of mechanization, high throughput screening methodologies, better scientific models, and powerful data analysis packages. Several water-saving technologies, for example, alternate wetting and drying (AWD) (Li, 2001; Tabbal et al., 2002), system of rice intensification (SRI) (Stoop et al., 2002), direct seeded aerobic rice (Bouman et al., 2002) have been proposed as suitable substitute to puddled system of rice cultivation (Kumar and Ladha, 2011).

Aerobic system of rice cultivation is growing as an economically feasible, water-labor-energy saving, mechanized, and climate smart agricultural practice to ensure food security. Seven to ten days early maturity of the aerobic rice crop compared to PTR allows timely planting of the succeeding crop in addition to the improvement in nutrient availability and soil conditions (Kumar and Ladha, 2011). Earlier aerobic rice varieties were developed with the aim to replace the low-yielding rice varieties of upland ecosystem (Nie et al., 2012). In the last decade, aerobic rice has not become popular among farmers due to high weed infestation and high cost to control weeds under aerobic situation as compared to PTR. With the availability of appropriate weed control measures, mechanization reducing labor requirements from 11 to 66% compared to PTR (Kumar et al., 2009; Rashid et al., 2009) and improved agronomic management practices; aerobic rice cultivation system is being successfully implemented in rainfed shallow lowland ecosystem.

In recent years, aerobic system of cultivation has gained momentum in irrigated lowlands where rainfall is not sufficient and pumping water from deep well is expensive, delta regions with delayed water supplies and upland system with supplemental irrigation. Aerobic rice needs 30–51% less total water for land preparation depending upon the soil types providing 32–88% higher crop productivity, 50% saving on labor (Wang et al., 2002) and can have 50% reduced GHG emission (Weller et al., 2016) compared to PTR (Bouman et al., 2005). The lack of stable yielding and dry direct seeded adapted varieties for aerobic system is a major limitation in achieving the maximum yield potential under water and resource limited conditions.

Most of the traits needed to improve yield in unfavorable environments are extremely complex in nature. Unraveling key regulators (QTLs/Genes) associated with grain yield and adaptability under aerobic conditions and combining the genes in the genetic background of high yielding mega rice varieties utilizing trait linked markers will ensure food security in the future. Molecular breeding can assist to carry precise breeding introgressing genes for tolerance to abiotic and biotic stresses needed for different regions of India.

The important key component of success in aerobic rice system is selection of widely adapted suitable aerobic rice cultivars (Wang et al., 2002). The present study was undertaken to identify the suitable aerobic adapted breeding lines suited to rainfed shallow lowland ecosystems of different countries of Asia; to study G (genotype) × E (environment) [genotype × location, genotype × season × location) interactions to identify genotypes that perform well over a broad range of growing conditions.

## Materials and Methods

### Plant Material and Experimental Sites

The plant material comprised of conventionally developed advanced breeding lines (genotypes) from the rainfed breeding program of IRRI which was developed through bi-parental (142 crosses) and complex cross (12 crosses). The advanced breeding lines were developed involving popular lowland rice varieties grown in different countries crossed with released/pre-breeding drought tolerant aerobic as well as shallow lowland suited lines developed at IRRI between 2009 and 2013 (Table 1). The generations were advanced by selfing and a total of 401 and 441 F_5_/F_6_ breeding lines were tested during wet season and dry season, respectively.

**Table 1:** Detailed description on number of crosses developed per seasons/years and parentage to develop advanced breeding lines used in the present study.

**Sr. No**.	**Year crossed**	**No. of crosses**		**Parentage**
		**Bi-parental cross**	**Complex cross**	
1	2009DS	5	1	IR04A212/IR08L119, IR 81896-B-B-182/Swarna, IR06A144/IR 55419-04, IR08L158/CNA 10657, IR 72889-46-3-2-1/IR 57514-PMI 5-B-1-2, **Sanhuangzhan No 2/IR 4630-22-2-5-1-3//Fedarroz 50/IRRI 146///IRRI 123/IR 45427-2B-2-2B-1-1//IR 77298-14-1-2-10/IR07F287**
2	2009WS	18	–	IR 70181-26-PMI 2-9-1-1/PSBRc 18, IR08L217/IR08L183, IR08L119/IR 64, IR08L183/MTU 1010, IR08L118/MTU 1010, Thadokkham1/IR 77298-14-1-2-10, Thadokkham1/IR08L119, IR10L411/IR11L261, IR08L217/IR08L183, IR10L128/IR09N520, IR08N158/IRRI 200, IR09L242/IR09L124, IR09N495/IRRI 200, IR08N158/IR10L105, IR09N520/IR10L105, IR 55419-04/MRQ 74, IR 78985-B-6-B-B-B/IR 78875-190-B-1-3, IR 77298-14-1-2-10/IR 77298-5-6-11
3	2010DS	24	–	IR 64/IR 84984-83-15-18-B-B-3, IR 71700-247-1-1-2/IR09L224, IR 72022-46-2-3-3-2/IR 77298-5-6-18, IR 72022-46-2-3-3-2/IR09L224, IR09L324/IR 78875-176-B-2, Aus 261/3*Swarna, IR 71700-247-1-1-2/IR 77298-5-6-18, IR 78985-B-6-B-B-B/IR 81063-B-94-U 3-1, IR 81039-B-173-U 3-3/IR 81063-B-94-U 3-1, IR10N102/IR 86931-B-400, IR09L324/IR 78908-143-B-4, IR09L332/IR 78875-176-B-2, IR 64/IR 84984-83-15-18-B-B, IR 84984-83-15-18-B-B-72/Anjali, IR 78985-B-6-B-B-B/IR07L276, IR 78985-B-6-B-B-B/IR07L225, IR 81039-B-173-U 3-3/IR 78875-190-B-1-3, IR 81039-B-173-U 3-3/IR07L276, IR 81039-B-173-U 3-3/IR07L225, IR 81039-B-173-U 3-3/IR08L126, IR07L270/IR 78875-190-B-1-3, IR07L270/IR 81063-B-94-U 3-1, IR07L270/IR07L276, IR07L270/IR08L126
4	2010WS	17	1	IR08N158/IR10L185, IR09N495/IR10L185, IR06N119/IR 57514-PMI 5-B-1-2, IR09N495/IR09L173, IR10L179/IR10L151, IR09L224/IR09L343, IRRI168/IR10L105, IR09L242/IR09L343, IR10L149/IR08N158, IR10L149/IR10L152, IR09N520/IRRI 200, IR10L128/IR08N128, IR09L179/IR10L105, IR10L128/IR05N173, IR09N495/IR10L105, IR10L178/IR10L128, IR06N119/IR09L173, **IR 77298-14-1-2-10/Q 74//IR 84984-83-15-18-B-B/Q 74**
5	2011DS	42	–	IR09L337/IR09L154, IR09L272/IR09L337, IR09L317/IR10L149, IR05N173/IR09L272, IR09L337/IR10L137, IR09L337/IR10L185, IR09L337/IRRI 200, IR09L337/IR10L178, IR09L337/IR10L184, IR09L337/IR09L348, IR09L337/IRRI 175, IR09L272/IR09L317, IR09L272/IR10L146, IR09L272/IR10L149, IR09L272/IR10L128, IR09L272/IR10L137, IR09L272/IR10L165, IR09L272/IR09L179, IR09L272/IR10L105, IR09L272/IR08L181, IR09L272/IR09L343, IR09L272/IR10L178, IR09L272/IR09L224, IR09L272/IR09L348, IR09L272/IR10L184, IR09L317/IR10L146, IR09L317/IR10L128, IR09L317/IR10L137, IR10L146/IR10L149, IR10L146/IR10L128, IR10L146/IR10L137, IR10L149/IR10L128, IR10L149/IR10L137, IR08N158/IR09L337, IR08N158/IR09L272, IR05N173/IR10L149, IR08N158/IR10L149, IR08N158/IR10L128, IR09N495/IR10L137, IR08N158/IR08L220, IR10L149/IR10L165, IRRI 176/IR11L184
6	2011WS	16	1	IRRI 176/IR11L184, IRRI 176/IR11L186, IRRI 176/IR11L261, IR11L108/IR09N534, IR11L230/IR04N106, IR11L186/IR09N534, IR11L269/IR08N121, IR11L269/IR09A228, IR11L259/IR07A253, IR11L147/IR07A260, IRRI 177/IR 72, IRRI 177/IR 87761-30-3-1-1, IR11L261/IR09A228, IR11L261/IR09A228, IR11L152/Sabitri, IRRI 149*3/IR 86918-B-305, **IRRI 154/MA ZHAN (RED)//IR08N194**
7	2012DS	5	7	IR07N112/IR11L186, IR08N120/IR10L182, IR08N120/IR11L269, IR 69428-6-1-1-3-3/IR11L101, IR 65600-81-5-3-2/IR11L147, **IR08L152/IR11L184//IR 71700-247-1-1-2, IR08L152/IR11L186//IR 71700-247-1-1-2**, **IR08L152/IR11L269//IR 71700-247-1-1-2, IRRI 176/IR11L226//IR 71700-247-1-1-2, IRRI 176/IR11L184//IR 71700-247-1-1-2, IRRI 176/IR11L269//IR 71700-247-1-1-2, IR09L179/IR11L269//IR 71700-247-1-1-2**
8	2012WS	1	–	IR10F188/KHO487-4
9	2013DS	14	2	IR 67962-84-2-2-2/IR08L216, IR 67966-44-2-3/Vandana, WAB 878-6-37-4-4-P2-HB/SARO 5, WAB 878-6-37-4-4-P2-HB/IRRI 148, IR 67966-44-2-3/IR08L216, IR 67966-44-2-3/IR 87707-446-B-B-B, IR09L179*2/B 1050 D-KN-1-1-1-1-3, TME 80518/CHAO MED NYAY, IR09L303/Glentendg, HHZ 8-SAL 6-SAL 3-Y2/IR09L204, HHZ 8-SAL 6-SAL 3-Y2/IR09L303, IR06N155/IR09L120, PR 37139-3-1-3-1-2-1/IR09L303, IR09L303*2/BENONG 130, **Dular (ACC 32561)/IRRI 148///IRRI 154/UPLRi 7//IR 87707-446-B-B-B/Kali Aus, IR08L181/Bulu Gendhah//IR 103569**

*DS, dry season; WS, wet season; crosses in bold letters are the complex crosses involving more than two parents*.

A series of 23 experiments were conducted under aerobic conditions at the International Rice Research Institute (IRRI, Los Baños), Philippines; Bangladesh Rice Research Institute (BRRI, Gazipur), Bangladesh; Regional Agriculture Research Station (RARS, Tarahara), Nepal; National Rice Research Program (NRRP, Hardinath), Nepal; and National Rice Research Institute (NRRI, Cuttack), India IRRI-South Asia Breeding Hub (SA), ICRISAT (Hyderabad), India; Rice Research Station (RRS), Kaul, Haryana (India) and National Agriculture and Forestry Research Institute (NAFRI), Lao PDR. The detailed description on experimental design, number of advanced breeding lines screened in each season per location and the management practices followed in each experiment and location is presented in Table 2.

**Table 2:** Details of the experiments conducted in 2014–17 across different ecosystems, seasons and years.

**Exp**	**Location**	**Year/Season**	**Experimental design**	**Date of sowing**	**No of rows**	**Row length (m)**	**Spacing (R-R × H-H) (cm)**	**No. of rep**	**No. of entries**	**Fertilizer rate (Kg)**
1	IRRI, Los Baños	2014DS	(28 × 4) AL	03/01/2014	8	4	20 × 20	3	112	100:40:40 N:P_2_O_5_:K_2_O
2		2014WS	RCBD	21/06/2014	8	4	20 × 20	3	32	80:35:35 N:P_2_O_5_:K_2_O
3		2015DS	(23 × 5) AL	17/01/2015	8	4	20 × 20	2	115	100:40:40 N:P_2_O_5_:K_2_O
4		2015WS	(12 × 5) AL	17/06/2015	8	4	20 × 20	2	60	80:35:35 N:P_2_O_5_:K_2_O
5		2016DS	(9 × 6) AL	08/12/2015	8	4	20 × 20	2	54	100:40:40 N:P_2_O_5_:K_2_O
6		2016WS	(41 × 4) AL	10/06/2016	8	4	20 × 20	2	164	80:35:35 N:P_2_O_5_:K_2_O
7		2017DS	(24 × 5) AL	15/12/2016	3	4	20 × 20	2	120	100:40:40 N:P_2_O_5_:K_2_O
8	BRRI, Gazipur	2015WS	(20 × 3) AL	05/07/2015	6	5.4	25 × 20	2	60	200:50:50:45:5 Urea:Triple Superphosphate:murate of potash:Gypsum:ZnSO_4_
9		2015DS	RCBD	28/11/2015	4	6	25 × 20	2	11	300:70:75:45:6 Urea:Triple Superphosphate:murate of potash:Gypsum:ZnSO_4_
10	BRRI, Rajshai	2016WS	(2 × 17) AL	02/07/2016	6	5.4	25 × 20	2	34	200:50:50:45:5 Urea:Triple Superphosphate:murate of potash:Gypsum:ZnSO_4_
11	NRRP,	2015DS	(4 × 8) AL	09/12/2014	5	5	20 × 20	2	32	90:30:30 N:P_2_O_5_:K_2_O
12	Hardinath	2015WS	(23 × 5) AL	01/07/2015	6	5	20 × 20	2	115	80:40:30 N:P_2_O_5_:K_2_O
13		2016DS	(7 × 4) AL	15/12/2015	5	5	20 × 20	2	28	90:30:30 N:P_2_O_5_:K_2_O
14	RARS, Tarahara	2015DS	(6 × 6) AL	09/12/2014	4	4	20 × 20	3	36	100:30:30 N:P_2_O_5_:K_2_O
15		2015WS	(23 × 5) AL	01/07/2015	6	5	20 × 20	2	115	90:30:30 N:P_2_O_5_:K_2_O
16		2016DS	(7 × 4) AL	15/12/2015	4	4	20 × 20	2	28	100:30:30 N:P_2_O_5_:K_2_O
17	NRRI, Cuttack	2015DS	(5 × 4) AL	12/01/2015	4	5	20 × 20	2	20	80:40:40 N:P_2_O_5_:K_2_O
18		2015WS	RCBD	08/07/2015	4	5	20 × 20	2	16	80:40:40 N:P_2_O_5_:K_2_O
19	IRRI SA Hub, Hyderabad	2015WS	(5 × 25) Augmented RCBD	04/07/2015	3	3	20 × 20	–	125	120:60:60 N:P_2_O_5_:K_2_O
20	RRS, Kaul	2015WS	(11 × 9) AL	04/07/2015	4	4	20 × 20	2	99	120:60:25 N:P_2_O_5_:ZnSO_4_
21		2016WS	RCBD	05/07/2016	4	4	20 × 20	2	9	120:60:25 N:P_2_O_5_:ZnSO_4_
22	NAFRI,	2015WS	RCBD	07/07/2015	4	4	20 × 20	3	126	90:30:30 N:P_2_O_5_:K_2_O
23	Lao PDR	2016DS	RCBD	03/01/2016	5	4	20 × 20	3	30	90:30:30 N:P_2_O_5_:K_2_O

*DS, dry season; WS, wet season; AL, alpha lattice; RCBD, randomized complete block design; rep: replications; R-R × H-H, row to row x hill to hill; m, meter (unit); cm, centimeter (unit); Kg, kilogram (unit)*.

### Agronomic Management Practices

#### Pedoclimatic Conditions

The Philippines is located at 14°10′11.81^′′^ N, 121°15′39.22^′′^ E, and the soil characteristics included pH 7.7 (0.01 M CaCl_2_), 39% clay, 21% sand, 36% silt, 16.0 meq 100 g^−1^ Ca, 7.8 meq 100 g^−1^ Mg, 1.09 meq 100 g^−1^ K, 20 mg kg^−1^ P, and 0.101% KjN. BRRI, Bangladesh is located at latitude 23°45′ N, longitude 90° 22′ E, elevation 8.4 m above sea level. The sand, silt and clay of the experimental site varied from 17 to 19, 59 to 63, and 21 to 22, respectively, soil porosity 41 to 50% and organic matter content varied from 0.58 to 2.13%. The NRRP, Hardinath is located at 26°47′46.5 ″N and 85°57′49.35″ E. The sand, silt, and clay content of the experimental site was 37, 42, and 20%, respectively. The pH value of the soil was near to neutral; pH = 6.59) and very low in available sulfur (0.79 ± 0.06 ppm) and available boron (0.37 ± 0.05 ppm). The organic matter (1.02 ± 0.007%), total nitrogen (0.07 ± 0.002 %), extractable potassium (42.49 ± 2.52 ppm), available zinc (0.83 ± 0.21 ppm), and available manganese (6.75 ± 0.64 ppm) status was also low. RARS, Tarahara is located at 26°42′16.85″ North latitude and 87°16′38.43″ East longitude, and at 136 meters above sea level. The climate is subtropical, the soil texture is dominated by clay loam, and soil pH ranges from 6.5 to 7.0. The texture of the soil was clay loam with 3.42% organic matter, 0.04%total nitrogen, 108 kg/ha available phosphorus and 133 kg/ha K. The soil of the experimental site at NRRI, cuttack was basically clay loam texture with 32% clay, 38% silt, and 33% sand with medium bulk density of 1.45 g/cm^3^. Nutritional analyses of the soil showed medium status with 0.53% organic carbon, 214.03 kg/ha available nitrogen, 36.15 kg/ha available phosphorus (P_2_O_5_), and 164.05 kg/ha available potassium (K_2_O). 85°55′48″ E to 85°56′48″ E longitudes and 20°26′35″ N to 20° 27′ 20″ N latitudes with the general elevation of the farm being 24 m above the sea level. The soil of the RRS, Kaul experimental site was basically clay loam, having 25% clay, 30% silt, and 45% sand with bulk density of 1.55 g/cm^3^. The soil was alkaline in reaction (pH 8.1), low in organic carbon (0.40%) and available nitrogen (190 kg/ha), medium in available phosphorus (36.6 kg/ha P_2_O_5_), and high in available potassium (371 kg/ha K_2_O). The Hyderabad is located at 17° 22′ 31″ N and 78° 28′ 27″ E and the soil at experimental site consists of 21% clay, >9% silt, and 72% sand with organic content 0.5 ± 1%. Lao PDR is located at 17.9757° N, 102.6331° E and the soil pH varied from 5 to 7.5.

#### Land Preparation

Land preparations under aerobic conditions involved plowing using terra disc plow (IRRI and BRRI), mold board plow (NRRP, RARS, and NRRI), disc plow (IRRI-SA), and harrow (NAFRI and RRS). The plowing was followed by 3 rotorvations at weekly intervals (IRRI and BRRI); 3 tilling with nine tine cultivator (NRRI, RARS); 2 tillering with cultivator, light irrigation, and single rotorvation after 3–4 days (NRRI); 3 rotorvations at alternate days (IRRI-SA); and planking (NAFRI and RRS) followed by field leveling. At IRRI and NRRI, the field was laser leveled and allowed for the first flush of weeds to emerge and grow for 3 weeks, then controlled with the application of glyphosate (1.0 Kg ai/ha).

#### Seed Rate

The seed rate varied from 20 to 30 Kg ha^−1^ (IRRI, BRRI, NRRI and RARS), 50 Kg ha^−1^ (NRRI), 30 to 40 Kg ha^−1^ (NAFRI), and 20 to 25 Kg ha^−1^ (RRS). Sowing was practiced using Wintersteiger plot seeder (IRRI), dibbling 2 to 3 seeds/hill (IRRI-SA and RRS Kaul), line sowing (NRRI) and seed drill (NRRI and RARS).

#### Insect/Pest/Weed Management

At IRRI, combination of pre-emergence [oxadiazon at 0.5 Kg ai ha^−1^ at 6 days after seeding (DAS)], early post emergence [bispyribac sodium 0.03 Kg ai ha^−1^ (9.7%, nominee) at 11 and 22 DAS] and spot weeding at 35 and 55 DAS was used to control weeds. Integrated pest management practices involving rat baiting using ditrac (0.05 g kg^−1^ = 0.005% brodifacoum) bait to control rats, pre-seeding application of fipronil (0.075 Kg ai ha^−1^) along the bunds and at 7 DAS along the plot edges was followed. At BRRI, pre-emergence herbicide (petilachlor 500 EC @ 1.0 Kg ai ha^−1^ at 6 days after seeding (DAS) was applied followed by hand weeding when needed. At NRRP, RARS, and NAFRI, application of pendimethaline @ 8 ml liter^−1^, nominee gold (bispyribac sodium) as post emergence @ 0.4 ml liter^−1^ plus one manual weeding was practiced to control weeds. At NRRI, combination of pre-emergence (oxadiazon @ 0.5 Kg ai ha^−1^ at 6 days after seeding (DAS), early post emergence [bispyribac sodium @ 0.03 Kg ai ha^−1^ (9.7%, nominee) between 15 and 20 DAS] and spot weeding at 35 DAS was used to control weeds. At IRRI-SA, combination of pre-emergence (pendimethalin @ 1.0 Kg ai ha^−1^ at 6 days after seeding (DAS), early post emergence [bispyribac sodium @ 0.03 Kg ai ha^−1^ (9.7%, nominee gold) at 11 and 22 DAS] and one hand weeding at 35 and 55 DAS was done to control weeds. Integrated pest management practices involving installing pheromone traps for controlling the adults of Yellow stem borer (*scirpophaga incertulus*) followed by chemical control by application of DuPont™ ferterra® (chlorantraniliprole 0.4% GR) @ 4 Kg ha^−1^ was practiced. At RRS, the field was irrigated after the sowing and pendimethelin @ 1.0 Kg ai ha^−1^ was applied as pre-emergence herbicide after 1 day of seeding and spot weeding at 35 DAS was used to control weeds. The leaf folder was controlled by application of cartap @ 7.5 Kg ha^−1^.

#### Irrigation

At IRRI, sprinkler method of irrigation was used during seedling establishment stage from 1 to 21 days after sowing (DAS) and thereafter surface irrigation was applied once or twice a week depending on weather and crop water status. The irrigated field was allowed to drain naturally through normal seepage and percolation. At NRRP, RARS, NRRI, IRRI-SA, and NAFRI, surface irrigation was applied twice a week or depending on weather and crop water status. The excess water was allowed to drain naturally and also through normal seepage and percolation.

#### Traits Measured

Across all locations days to 50% flowering (DTF) was recorded when ~50% of the plants in a plot had shown panicle exertion. At maturity, the plant height (PHT) of the randomly selected three plants per plot was measured from the base of the plant to the tip of the highest panicle of the plant using centimeter scale. At maturity, the harvested grains per plot were first threshed and then oven dried for 3 days at a temperature set of 50°C. The grain yield (GY) was calculated after normalizing the plot yield to a moisture content of 14%.

#### Genotyping of Promising Lines

The polymorphic simple sequence repeats (SSRs) markers in the earlier reported QTL region for anaerobic germination (Angaji et al., 2010), early vegetative vigor, nodal roots, root hair length, root hair density, early and uniform emergence, and grain yield under direct seeding (Sandhu et al., 2015), drought tolerance (Bernier et al., 2007; Venuprasad et al., 2009; Vikram et al., 2011) and gene-specific markers for blast, bacterial blight, and gall midge were identified. The details on the markers used to identify the QTLs/gene is presented in Supplementary Table 1.

### Statistical Analysis

#### Season-Wise Two Stage Analysis

For each season, in the first stage, individual trials (location × season) are analyzed using a mixed model that considers genotypes as fixed and replicate and block within replicate effects as random. Trial-wise broad-sense heritability (H) was calculated as:

$$\begin{array}{l} \text{H}=\frac{{\sigma^{2}}_{g}}{{\sigma^{2}}_{g}+\frac{{\sigma^{2}}_{e}}{r}} \end{array}$$

where $\sigma_{g}^{2}$ represents the genotypic variance, $\sigma_{e}^{2}$ the error variance, and *r* represents the number of total replications. The first stage analysis produced the adjusted means for the genotypes in environment *j* having estimated variance-covariance matrix *Vj*. If adjusted means are sorted by environments (by location, year) and then by genotypes within each environment, the variance covariance matrix across trials “*V*” is of the block-diagonal form

$$\begin{array}{l} V=\;{⊕}_{e=1}^{mn}V_{e}=\left(\begin{array}{cccc} V_{11} & 0 & \ldots & 0\\ 0 & V_{22} & \ldots & 0\\ \vdots & \vdots & \ddots \; & \vdots \\ 0 & 0 & \ldots & Vmn \end{array}\right) \end{array}$$

where ⊕ is the direct sum operator, *m* is the number of sites and *n* is the number of years, and *V*_*jk*_ is the estimated variance-covariance matrix of adjusted means in the jth environment. The weighting matrix is given by D(${V_{jk}}^{-1}$), where D(${V_{jk}}^{-1}$) is a diagonal matrix with diagonal elements equal to those of ${V_{jk}}^{-1}$ (Smith et al., 2001, 2005). At the second stage, the marginal means for genotypes across environments are obtained using a mixed model is fitted to the table of adjusted means by fixing the error variance. The mixed model is given by

$$\begin{array}{l} \hat{Y_{ijk}}=\mu +g_{i\;}+y_{k\;}+gy_{ik}+l_{j}+ly_{jk}+gl_{ij}+gyl_{ijk}+\epsilon_{ijk}\hat{Y_{ijk}} \end{array}$$

is the adjusted mean of the ith genotype in the jth location and kth year, μ is the overall mean, *g*_*i*_ is the main effect of the ith genotype, *l*_*j*_ is the main effect of the jth location, *y*_*k*_ is the main effect of the kth year, *ly*_*jk*_ is the interaction of the jth location with the kth year, *gy*_*ik*_ is the interaction of the ith genotype with the kth year, *gl*_*ij*_ is the interaction of the ith genotype with the jth location, *gyl*_*ijk*_ is the interaction of the ith genotype with the jth location in the kth year, ϵ_*ijk*_ is the error. The error variance matrix will be a diagonal matrix. The error variance matrix is given by D(Ve^−1^), where D (Ve^−1^) is a diagonal matrix with diagonal elements equal to those of Ve^−1^. The residual variance is set to unity. The model is fitted using PROC HPMIXED. HPMIXED is suitable when there are a large number of observations, or a large number of random effects or large number of levels of fixed effects like in the present case. Damesa et al. (2017) provide the SAS macro to calculate the weights proposed by Smith et al. (2001) and for the second stage of the two-stage analysis.

#### Yield Stability Analysis

A total of 48 advanced breeding lines that were tested in five or more than five trials were included in the yield stability analysis. The location × year × season combination is now considered as an “environment (E).” Stability was assessed using the three generally used models, (i) Shukla stability variance model (Shukla, 1972), (ii) Eberhart–Russell model (Eberhart and Russell, 1966), and (iii) Finlay-Wilkinson model (Finlay and Wilkinson, 1963) and the AMMI-1 (Additive Main effects and Multiplicative model with one multiplicative term) (Gauch, 1988, 1992). The best-fitting model was chosen based on the lowest AIC (Akaike Information Criterion) value. The stability models were fitted into the genotype × environment means within a mixed-model framework where the effect of the genotypes was considered as fixed and the trials were random (Piepho, 1998, 1999; Raman et al., 2011).

## Results

### Trial-Wise Means and Heritability

At IRRI, under the aerobic condition the mean DTF varied from 66 to 83 days, the mean plant PHT varied from 97 to 129 cm and mean GY from 4,035 to 6,433 Kg ha^−1^. The heritability's (H) of the trials for GY varied from moderate to high (0.54 to 0.86) at IRRI (Table 3). In Bangladesh, the mean DTF ranged from 86 to 99 days, PHT from 93 to 112 cm, and mean grain yield varied from 2,528 to 5,064 Kg ha^−1^ with estimated heritability for the grain yield ranging from 0.78 to 0.90. In Nepal, the phenotypic variability under aerobic conditions varied from 75 to 92 days for DTF, 95 to 120 cm for mean PHT and 2,085 to 4,793 Kg ha^−1^ for mean grain yield (Table 3). In India, the variability for mean DTF ranged from 67 to 95 days, the mean PHT from 93 to 112 and mean GY from 3,300 to 4,712 Kg ha^−1^. At NAFRI, Lao PDR, under aerobic condition the DTF ranged from 82 to 89 days, mean PHT from 89 to 98 cm and mean grain yield 3,315 to 3,525 Kg ha^−1^. The estimated broad sense heritability for grain yield ranged from 0.48 to 0.78, 0.65 to 0.90, and 0.59 to 0.71 in Nepal, India and Lao PDR, respectively (Table 3).

**Table 3:** Descriptive agronomic traits statistics across different ecosystems, seasons, and years under aerobic conditions.

**Exp**	**Location**	**Year/Season**	**Trial mean/LSD**						**Trial H**
			**DTF (days)**	**LSD (0.05%)**	**PHT (cm)**	**LSD (0.05%)**	**GY (Kg ha^**−1**^)**	**LSD (0.05%)**	**GY**
1	IRRI (Los Baños)	2014DS	77	3.42	110	7.92	4,833	972	0.74
2	Philippines	2014WS	66	1.89	129	8.75	4,417	914	0.86
3		2015DS	83	4.6	97	10.74	4,644	1,014	0.54
4		2015WS	73	3.0	110	9.40	5,292	984	0.74
5		2016DS	70	2.4	97	8.74	6,433	912	0.64
6		2016WS	75	1.3	103	3.36	4,035	828	0.78
7		2017DS	79	2.87	107	10.04	5,290	1,060	0.52
8	BRRI (Gazipur)	2015DS	93	1.56	93	3.72	5,064	285	0.90
9	Bangladesh	2015WS	86	2.40	112	7.21	2,528	506	0.78
10	BRRI (Rajshahi) Bangladesh	2016WS	99	0.91	108	2.49	2,870	242	0.88
11	NRRP, (Hardinath)	2015DS	75	7.33	120	10.68	4,018	1,183	0.69
12	Nepal	2015WS	83	11.12	112	11.53	4,793	1,301	0.48
13		2016DS	91	5.49	95	10.57	2,530	538	0.60
14	RARS (Tarahara),	2015DS	75	3.31	107	6.99	4,730	663	0.59
15	Nepal	2015WS	76	5.89	99	10.50	2,567	858	0.68
16		2016DS	92	13.00	107	9.32	2,085	800	0.78
17	NRRI (Cuttack),	2015DS	67	2.08	101	2.13	3,843	123	0.86
18	India	2015WS	70	1.76	103	1.98	4,012	840	0.87
19	IRRI SA Hub (Hyderabad), India	2015WS	74	4.90	95	12.13	4,423	1,024	0.90
20	RRS (Kaul) India	2015WS	95	2.87	93	4.22	3,300	440	0.65
21		2016WS	76	3.25	112	3.68	4,712	520	0.72
22	NAFRI, Lao PDR	2015WS	89	5.82	89	8.20	3,525	834	0.59
23		2016DS	82	9.53	98	9.61	3,315	608	0.71

*DS, dry season; WS, wet season; DTF, days to 50% flowering (days); PHT, plant height (cm); GY, grain yield (Kg ha^-1^); H, heritability*.

### Season-Wise Two Stage Analysis

The analysis indicated significant genotype × location interaction for yield and PHT under both wet and dry seasons. The genotype x season interaction was non-significant for all traits in the case of dry season and 0 for all traits in wet season trials. The genotype × season × location interaction for yield was non-significant in both seasons indicating that at each location the genotypes did not significantly vary in their performances across seasons (Table 4). The genotype × season × location interaction for DTF was significant in dry season.

**Table 4:** REML parameters of the two-stage analysis across the locations and years for each season.

**Effect**	**Wet season**			**Dry season**		
	**Grain yield**	**Days to 50% flowering**	**Plant height**	**Grain yield**	**Days to 50% flowering**	**Plant height**
Season	0.184 (NS)	0.5895 (NS)	14.8938 (NS)	0	1.239 (NS)	0
Location	1.456 (NS)	23.4317 (NS)	29.2409 (NS)	0.4097 (NS)	50.065 (NS)	22.0889 (NS)
Season^*^location	0.0592 (NS)	0	54.4876 (NS)	0.9108^*^	11.603 (NS)	31.6585 (NS)
Genotype^*^Season	0	0	0	0.01473 (NS)	0.00011 (NS)	1.0329 (NS)
Genotype^*^Location	0.1077^*^	28.1893^**^	23.5047^**^	0.1954^**^	4.1269 (NS)	3.3838^*^
Genotype^*^Season^*^Location	0.0596 (NS)	2.3521 (NS)	2.2099 (NS)	0.02435 (NS)	13.6596^**^	0
Residual	1	1	1	1	1	1

* indicates significance at 5% level.

** indicates significance at 1% level.

*NS, non-significance*.

The means of entries tested in 3 or more years for these traits are shown in Table 5. Among genotypes tested in four or more trials the best five performers in both the seasons were IR 97057-10-1-1-2, IR 91328-43-6-2-1, IR 91326-19-2-1-2, IR 92521-146-3-3-2, IR 91326-7-13-1-1, IR 97041-8-1-1-1. The best performers in wet season that were tested in five or more trials were IR 91326-19-2-1-2, IR 92521-146-3-3-2, IR 91328-43-6-2-1, IR 91326-7-13-1-1, and IR 97041-8-1-1-1 (Table 5). The best performers in dry season that were tested in five or more trials were IR 92521-172-5-1-1, IR 97041-8-1-1-1, IR 91328-43-6-2-1, IR 92521-146-3-3-2, and IR 92521-173-1-3-2 (Table 5).

**Table 5:** Mean performances of lines tested in 3 or more trials (site × year) for days to 50% flowering (days), plant height (cm), and grain yield (Kg ha^−1^) across years and location.

**Wet season**								**Dry season**							
**Genotype**	**NOT**	**GYKGPHA**	**std. error**	**DTF**	**std. error**	**PHT**	**std. error**	**Genotype**	**NOT**	**GYKGPHA**	**std. error**	**DTF**	**std. error**	**PHT**	**std. error**
IR 91326-20-2-1-2	7	3400	0.63	71	3.67	111	4.98	IR 91326-19-2-1-2	8	4420	0.48	74	3.27	100	3.76
IR 91328-43-6-2-1	7	3720	0.63	71	3.67	110	4.98	IR 93835-70-2-2-1	8	3380	0.48	73	3.27	108	3.76
IR 92521-173-1-3-2	7	3640	0.63	71	3.67	110	4.98	IR 93835-73-2-3-1	8	3480	0.48	71	3.27	107	3.76
IR 91326-19-2-1-2	6	3950	0.65	69	4.12	107	5.21	IR 91328-43-6-2-1	7	4540	0.49	75	3.32	99	3.84
IR 92521-143-2-2-1	6	3350	0.63	72	3.68	110	4.99	IR 91326-20-2-1-2	5	4230	0.53	73	3.32	106	4.11
IR 92521-146-3-3-2	6	3850	0.65	71	4.12	104	5.21	IR 91326-7-13-1-1	5	4290	0.51	74	3.29	100	4.05
IR 93835-70-2-2-1	6	3670	0.65	71	4.12	115	5.21	IR 92521-146-3-3-2	5	4420	0.53	73	3.32	101	4.11
IR 91326-7-13-1-1	5	4020	0.64	70	3.69	109	5.04	IR 92521-173-1-3-2	5	4360	0.53	71	3.30	101	4.03
IR 93835-133-3-1-1	5	3370	0.65	70	4.13	120	5.22	IR 92521-172-5-1-1	5	4660	0.51	72	3.29	102	4.05
IR 93835-167-2-2-1	5	3620	0.65	69	4.13	128	5.22	IR 92521-120-1-3-2	5	4280	0.51	72	3.29	104	4.05
IR 93835-73-2-3-1	5	3690	0.65	69	4.13	118	5.25	IR 93823-36-1-1-1	5	3860	0.51	72	3.29	103	4.05
IR 93835-74-3-1-1	5	3350	0.65	69	4.13	120	5.22	IR 93835-74-2-1-1	5	3610	0.51	69	3.28	109	4.02
IR 97041-8-1-1-1	5	3720	0.65	82	4.14	103	5.26	IR 97041-8-1-1-1	5	4550	0.52	83	3.60	97	4.08
IR 97043-33-1-1-3	5	3650	0.65	73	4.14	107	5.26	IR 91326-20-2-1-4	4	4300	0.54	74	3.34	102	4.23
IR 97048-10-1-1-3	5	3210	0.65	80	4.14	110	5.26	IR 92521-143-2-2-1	4	4640	0.51	73	3.27	102	4.01
IR 91326-20-2-1-4	4	3670	0.66	71	4.15	109	5.30	IR 92521-173-1-1-1	4	4800	0.51	73	3.28	100	4.13
IR 92521-114-2-2-2	4	3730	0.69	70	4.51	106	5.34	IR 92521-114-2-2-2	4	4410	0.52	73	3.30	102	4.17
IR 92521-172-5-1-1	4	3560	0.66	73	4.15	106	5.30	IR 92521-143-4-3-2	4	4290	0.54	74	3.34	100	4.23
IR 92521-143-4-3-2	4	3600	0.66	71	4.15	105	5.30	IR 92521-143-7-2-1	4	4470	0.54	75	3.34	106	4.23
IR 92521-143-7-2-1	4	3450	0.66	72	4.15	109	5.30	IR 92521-147-3-1-2	4	4540	0.52	74	3.30	103	4.17
IR 92521-120-1-3-2	4	3590	0.66	73	4.15	103	5.30	IR 92521-114-6-3-1	4	4600	0.79	79	3.84	100	6.02
IR 93823-36-1-1-1	4	3410	0.66	69	4.15	115	5.30	IR 92521-143-3-1-2	4	5100	0.79	76	3.84	107	6.02
IR 93835-74-2-1-1	4	3080	0.66	69	4.15	120	5.30	IR 95809-1-1-2-2	4	4040	0.54	82	3.74	99	4.36
IR 97036-15-2-1-1	4	4050	0.66	82	4.17	114	5.32	IR 93821-41-1-2-1	4	3980	0.52	71	3.30	109	4.17
IR 97041-5-1-1-2	4	4120	0.66	74	4.17	106	5.32	IR 93828-195-2-1-1	4	3840	0.52	70	3.30	114	4.17
IR 97043-20-2-1-1	4	3420	0.66	78	4.17	109	5.32	IR 93835-133-3-1-1	4	3430	0.54	72	3.34	113	4.23
IR 97043-31-2-1-2	4	3530	0.66	74	4.17	107	5.32	IR 97041-5-1-1-2	4	3700	0.53	81	3.66	96	4.19
IR 97043-33-4-1-1	4	3150	0.66	77	4.17	111	5.32	IR 97048-10-1-1-3	4	5060	0.53	80	3.66	98	4.19
IR 97046-42-2-1-2	4	3620	0.66	78	4.17	105	5.32	IR 97057-10-1-1-2	4	4330	0.53	78	3.66	101	4.19
IR 97057-10-1-1-2	4	4130	0.66	73	4.17	113	5.32	IR 97062-36-1-1-1	4	4400	0.53	83	3.66	92	4.19
IR 97062-36-1-1-1	4	3730	0.66	77	4.17	104	5.32	IR 97071-20-1-1-3	4	5050	0.53	85	3.66	100	4.19
IR 97071-14-2-1-2	4	3530	0.66	75	4.17	110	5.32	IR 91326-20-1-1-1	4	4560	0.51	74	3.27	103	4.10
IR 97071-20-1-1-3	4	3980	0.66	79	4.17	113	5.32	IR 97043-33-1-1-3	4	4370	0.53	78	3.63	99	4.19
IR 83399-B-B-52-1	3	4180	0.69	73	4.83	115	5.61	IR 91326-20-1-1-2	3	4310	0.55	72	3.36	104	4.40
IR 91326-20-1-1-1	3	3640	0.69	72	4.51	108	5.34	IR 95815-4-1-1-2	3	4100	0.54	84	3.74	91	4.36
IR 92521-173-1-1-1	3	3490	0.69	71	4.51	105	5.34	IR 95783-6-2-2-3	3	4670	0.54	80	3.74	97	4.36
IR 97046-39-2-1-2	3	3290	0.69	80	4.17	103	5.76	IR 95809-50-1-1-1	4	3690	0.51	80	3.43	95	4.04
IR 97093-6-2-2-2	3	2700	0.69	80	4.17	100	5.76	IR 95814-37-1-2-2	3	3850	0.54	83	3.74	94	4.36
IR 93340:34-B-2-B-2-1	3	3550	0.69	77	4.17	103	5.71	IR 95840-33-3-2-3	3	4330	0.54	83	3.74	97	4.36
IR 64^*^	4	2820	0.73	78	4.59	102	5.91	IR 95842-21-1-1-3	3	4360	0.54	85	3.74	92	4.36
Vandana^*^	4	3460	0.72	72	5.81	107	5.75	IR 95862-10-5-1-3	3	4040	0.54	78	3.74	112	4.36
IRRI 132^*^	3	3430	0.73	76	4.48	111	5.68	IR 93835-167-2-2-1	3	3450	0.57	70	3.54	126	4.59
IRRI 148^*^	3	2930	0.82	75	5.81	111	6.74	IR 93835-74-3-1-1	3	3690	0.57	72	3.54	113	4.59
UPL Ri 7^*^	3	4160	0.82	77	5.81	110	6.74	IR 98786-13-1-2-1	3	4150	0.54	82	3.81	108	4.38
Ghaiya-1^*^	2	3380	0.76	87	4.79	96	6.07	IR 98869-19-1-1-1	3	4660	0.54	82	3.74	110	4.36
Hardinath-1^*^	2	3890	0.76	81	5.04	106	6.49	IR 98959-19-1-1-1	3	4340	0.54	81	3.74	92	4.36
Hardinath-2^*^	2	3920	0.76	80	5.04	111	6.49	Vandana^*^	7	4090	0.51	77	3.21	104	3.83
IRRI 119^*^	2	1570	0.77	86	4.69	107	6.30	UPL Ri 7^*^	6	4080	0.54	83	3.28	105	4.03
IRRI 123^*^	2	2530	0.77	82	4.65	99	6.24	IRRI 132^*^	4	4150	0.55	85	3.34	103	4.23
Sukkhadhan-3^*^	2	2830	0.76	91	5.04	117	6.49	NSIC Rc 192^*^	4	4660	0.52	76	3.37	103	4.21
Sukkhadhan-4^*^	2	3190	0.76	67	5.04	112	6.49	IR 64^*^	3	4240	0.69	80	3.60	91	4.83
Swarna^*^	2	2060	0.77	103	6.78	78	8.04	IRRI 123^*^	2	3480	0.72	82	3.57	89	5.38
Tarahara-1^*^	4	2360	0.76	82	5.04	105	6.49	IRRI 148^*^	2	3910	0.72	75	3.64	105	5.09
BRRI dhan56^*^	3	3580	0.92			109	6.76	Katihan1^*^	2	4700	0.73	78	3.83	104	5.98
BRRI dhan57^*^	3	3010	0.92			103	6.76	Swarna^*^	2	3240	0.72	99	3.57	65	7.34
BRRI dhan66^*^	3	4230	0.94	78	6.10	109	6.79	Sabitri^*^	4	2680	0.98	98	4.68	82	7.34
BRRI dhan71^*^	3	4860	0.94	79	6.10	111	6.79	Sahabahgi Dhan^*^	6	4130	0.72	85	4.02	92	5.98
IR 13L491^*^	2	4120	0.92	79	6.01	124	7.37	Sahod Ulan-1^*^	6	4090	0.86	76	3.81	97	6.56
MTU 1010^*^	4	2110	1.00	82	6.78	107	8.04	Samba Mahsuri^*^	4	4260	0.98	100	4.68	73	7.34
NSIC Rc192^*^	5	3690	0.92			109	7.10	Tarahara-1^*^	2	4090	0.68	80	3.69	107	5.49
Samba Mahsuri^*^	5	1150	0.99	111	6.78	99	8.04	TDK11^*^	2	4280	0.68	76	5.00	116	6.37
Sukkhadhan-5^*^	6	3280	0.99	92	6.78	100	8.04								
Sukkhadhan-6^*^	6	2050	0.99	89	6.78	106	8.04								
TDK11^*^	2	2180	0.99	85	6.78	119	8.04								
TDK8^*^	2	2150	0.99	85	6.78	112	8.04								

NOT, number of trials the genotype has been tested; GYKGPHA, grain yield Kg ha^-1^; std. error, standard error; DTF, days to 50% flowering; PHT, plant height.

* *Check varieties*.

### Stability Analysis

AMMI-1 model was the best fitting stability model (AIC = 542.6). Entries IR 97046-42-2-1-2, IR 93835-167-2-2-1, and IR 97041-5-1-1-2 and the checks IRRI 132 and UPLRi 7 had relatively large factor loadings indicating higher sensitivity to changing environmental conditions. Of these the best yielder was IR 97046-42-2-1-2 (4,346.14 Kg ha^−1^ during wet season and 2,754.22 Kg ha^−1^ during dry season across five trials). The best performers were IR 92521-143-7-2-1, IR 93835-74-2-1-1, IR12L355, IR 97043-33-4-1-1 and IR 97041-8-1-1-1. Of this IR12L355 yielded 4174.34 Kg ha^−1^ during wet season and 3,830.73 Kg ha^−1^ during dry season.

The second-best model was the Finlay-Wilkinson model (AIC = 602.3). Based on the Finlay-Wilkinson regression model, a regression coefficient approximating to the value of 1.0 indicates an average stability. When it is allied with high yield, the genotype possesses general adaptability and when allied with low mean yield, the genotype is poorly adapted to all studied environments. This includes entries IR 92521-172-5-1-1, IR 93835-70-2-2-1, IR 92521-146-3-3-2, IR 91326-20-1-1-2, and IRRI 148. Of these IRRI 148 was moderately good yielding, 3,552.42 Kg ha^−1^ during wet season and 3,160.87 Kg ha^−1^ dry season. Note that IRRI 148 was tested in five trials at IRRI only. IR 93835-70-2-2-1 yielded 3,550.98 Kg ha^−1^ and 3,001.65 Kg ha^−1^ in wet and dry season respectively and IR 97041-8-1-1-1 yielded 3374.60 Kg ha^−1^ and 2,904.00 Kg ha^−1^ in wet and dry season, respectively.

Regression coefficient value (rescaled beta) provides estimation about the adaptability of genotype across different environments and locations and helps plant breeders to select the high yielding stable genotypes. Regression coefficient increasing above the value of 1.0 for the genotypes IR 92521-173-1-3-2, IR 93835-70-2-2-1, IR 91326-20-2-1-2, IRRI 132, and IR 97041-8-1-1-1, represents the adaptability of these genotypes to high yielding environment with more sensitivity to the environmental change (Fageria, 1992). Regression coefficients decreasing below the value 1.0 (Fageria, 1992) for the genotypes IR 92521-143-7-2-1, IR 92521-143-2-2-1, and IR 97043-33-4-1-1 indicates high resistance to the change in environment with adaptability to low-yielding environments.

### QTLs for Biotic and Abiotic Stress Tolerance/Resistance Present in the Promising Lines

The selected promising aerobic rice genotypes were screened for the presence of QTLs/gene increasing rice adaptability to aerobic condition such as- anaerobic germination, early vegetative vigor, nodal roots, root hair length, rot hair density, early and uniform emergence, and grain yield under direct seeding (Dixit et al., 2015; Sandhu et al., 2015), drought tolerance (Bernier et al., 2007; Khowaja et al., 2009; Venuprasad et al., 2009, 2012; Vikram et al., 2011) and for resistance/tolerance to blast (Fjellstrom et al., 2004; Qu et al., 2006; Koide et al., 2011; Shikari et al., 2013) bacterial blight (Song et al., 1997; Chu et al., 2006; Swamy et al., 2006; Perumalsamy et al., 2010; Ullah et al., 2012), brown plant hopper (Sun et al., 2005; Jairin et al., 2007), and gall midge (Nair et al., 1996; Sama et al., 2012). Among the earlier identified QTLs/genes *AG*_*9.1*_*, qEMM*_*1.1*_ *qNR*_*5.1*_*, qEVV*_*9.1*_*, qRHD*_*1.1*_*, qRHD*_*5*_*, qRHD*_*8.1*_*, qGY*_*6.1*_*, qDTY*_*2.1*_*, qDTY*_*3.1*_*, qDTY*_*12.1*_*, BPH3, BPH17, GM4, xa4, Xa21, Pita, and Pita2*, were identified to be present in the breeding lines providing higher yield and adaptability under aerobic conditions. Among the identified QTLs, the frequency of *xa4* gene was highest followed by *qAG*_*9.1*_, *GM4, qDTY*_*3.1*_*, qDTY*_*2.1*_*, qGY*_*6.1*,_ and *qDTY*_*12.1*_. Among the breeding lines highest number of QTLs were identified in IR 92521-143-7-2-1 and IRRI 148 with 8 QTLs, followed by IR 92521-172-5-1-1 with 7 QTLs and seven genotypes possessing 6 QTLs. It is also evident from the study that *qDTY*_3.1_ either single or in combination with *qDTY*_12.1_ and/or *qDTY*_2.1_, *qGY*_6.1_ provide grain yield stability across different locations under aerobic conditions.

## Discussion

Aerobic system of rice cultivation is an efficient, mechanized, resourceful and economically viable alternative to PTR cultivation system. Its benefits include saving of water-labor and energy (Tuong et al., 2005), improved soil physical conditions (Buresh and Haefele, 2010), timely establishment of succeeding crop, reduce GHG emission (Weller et al., 2016), increased agricultural productivity, and environmental sustainability (Bouman et al., 2005). Now a day's aerobic system of rice cultivation is becoming popular in South Asia (India, Bangladesh and Nepal), South East Asia (Cambodia, Myanmar, Lao PDR, Philippines, and Vietnam) and to some extent in the West Africa (Kumar and Ladha, 2011).

Genotype x environment (G × E) interaction arises when different genotypes react differently to the different environments and are paramount in the identification and development of genotypes that perform well over a wide range of growing conditions (Malosetti et al., 2013; Dou et al., 2016). The adjusted mean of genotypes over the environments based on the combined analysis of variance is used to select genotypes that are superior across the test environments and are good performers in comparison with the checks that have a general good adaptability. There were either significant genotype × location or genotype × season × location interactions for the agronomic traits including days to 50% flowering, plant height, and grain yield under aerobic conditions. The existing variability may be due to the variability in topography (Peng et al., 2006), soil types, fertility and organic matter turn over, soil nutrient dynamics (Gao et al., 2006), water regime, nutrient cycle, nutrient availability, and uptake (Belder et al., 2005; Nie et al., 2008; Kreye et al., 2009). Best genotypes in terms of high mean were identified for the wet (WS) and dry seasons (DS) separately (Table 5). Across seasons, the genotypes showed variable grain yield except some genotypes.

General adaptability is a criterion for selecting genotypes, however it is necessary to assess the stability of their performances over varying environmental conditions in large area testing such as the present. Assessing the yield stability has mainly been centered on the analysis of G × E interaction in the cultivar trials. The well-known stability measures can be expressed as parameters of a general mixed model by modeling the covariance structure of G × E interactions (Piepho, 1998). The stable genotypes identified by the two best models were different (Table 6). IR 91326-19-2-1-2 and IR 92521-146-3-3-2 identified by the AMMI-1 were good yielders. IR 97041-8-1-1-1 was identified as a stable genotype by both models (Table 6).

**Table 6:** Promising lines, their stability ranking based on the best fitting models and the mean yield (kg ha^−1^) of stable genotypes and QTLs present in promising genotypes increasing rice adaptation under aerobic situation and resistance/tolerance to biotic stresses.

**ENTRY**	**Estimate**	**Std Error**	**NOL**	**WS**	**DS**	**rank**	**QTLs/gene**
**AMMI(1) MODEL**							
IR 92521-143-7-2-1	0.0435	0.5483	5	3358.3	2707.8	1	*AG_*9.1*_+GM4+xa4+qEMM_*1.1*_ +qGY_*6.1*_+qDTY_*2.1*_+qDTY_*3.1*_* *+qDTY_*12.1*_*
IR 93835-74-2-1-1	0.1284	0.5273	5	2719.5	2401.6	2	*BPH3+GM4+xa4+qRHD_*5.1*_ +qDTY_*3.1*_*
IR 91326-19-2-1-2	0.1802	0.5077	6	4174.3	3830.7	3	*AG_*9.1*_+GM4+xa4+qDTY_*2.1*_ +qDTY_*3.1*_+qDTY_*12.1*_*
IR 97043-33-4-1-1	0.2296	0.5352	5	3027.9	2520.1	4	*BPH17+GM4+xa4+Xa21 +qEMM_*1.1*_+qRHD_*1.1*_*
IR 97041-8-1-1-1	0.2308	0.5316	6	3374.6	2904.0	5	*AG_*9.1*_+GM4+xa4+Xa21+Pita* *+qNR_*5.1*_*
IR 92521-146-3-3-2	0.2701	0.5137	7	4323.4	4048.4	6	*AG_*9.1*_+GM4+xa4+qDTY_*12.1*_* *+qDTY_*3.1*_*
IR 92521-172-5-1-1	0.9955	0.3164	6	3589.7	2490.7	7	*AG_*9.1*_+GM4+xa4+qEMM_*1.1*_ +qGY_*6.1*_+qDTY_*2.1*_* *+qDTY_*3.1*_*
**FINLAY-WILKINSON MODEL**							
IR 92521-172-5-1-1	0.9955	0.3164	6	3589.7	2490.7	1	*AG_*9.1*_+GM4+xa4+qEMM_*1.1*_ +qGY_*6.1*_+qDTY_*2.1*_+qDTY_*3.1*_*
IR 93835-70-2-2-1	1.0189	0.3277	6	3551.0	3001.7	2	*BPH3+xa4+qGY_*6.1*_+qDTY_*3.1*_* *+qRHD_*5.1*_+qEVV_*9.1*_*
IR 92521-146-3-3-2	0.9777	0.3125	8	3443.9	2873.1	3	*AG_*9.1*_+GM4+xa4+qDTY_*12.1*_ +qDTY_*3.1*_*
IR 91326-20-1-1-2	1.0234	0.329	8	3267.4	2901.0	4	*AG_*9.1*_+GM4+xa4+qDTY_*2.1*_ +qDTY_*3.1*_+qDTY_*12.1*_*
IRRI 148	1.0244	0.6142	5	3552.4	3160.9	5	*AG_*9.1*_+GM4+xa4+qEMM_*1.1*_ +qGY_*6.1*_* *+qDTY_2.1_+qDTY_3.1_+qDTY_12.1_*
IR 91326-19-2-1-2	0.9568	0.3153	6	3263.6	2815.1	6	*AG_*9.1*_+GM4+xa4+qDTY_*2.1*_ +qDTY_*3.1*_+qDTY_*12.1*_*
IR 97041-8-1-1-1	0.9564	0.3155	6	3374.6	2904.0	7	*AG_*9.1*_+GM4+xa4+Xa21+Pita +qNR_*5.1*_*

*WS, wet season; DS, dry season; rank, stability rank; NOL, the number of locations in which the entry was tested*.

In practice, there are not many successful examples of systematic aerobic rice breeding programs. Most of the varieties used for aerobic rice cultivation are the varieties that have been developed for upland conditions (Zhao et al., 2010). The lack of stable high yield appropriate rice varieties is the major limitations to achieve the maximum yield potential under aerobic system of rice cultivation. Mean grain yield of upland adapted rice varieties ranged from 3.0 to 8.8 t ha^−1^ has been observed under aerobic rice cultivation system (George et al., 2002; Bouman et al., 2005, 2007; Xiaoguang et al., 2005; Atlin et al., 2006; Pinheiro et al., 2006; Feng et al., 2007). The mean GY across locations, seasons and years under aerobic condition was 5.0 t ha^−1^, indicating the high grain yield potential and adaptability of these genotypes across different ecosystem.

The stable good yielding genotype IR 91326-19-2-1-2 (IR 06A144/IR 55419-04) showed 11% higher yield over Vandana (Philippines), 14.4% over BRRIdhan 29 (Bangladesh), 11.3% over Hardinath, and 3% over Tarahara (Nepal) (Table 7). IR 97041-8-1-1-1 (IRRI 105/IR 78877-163-B-1-1//IR 78878-53-2-2-2/IR03A550) identified as stable genotype by both models showed yield advantage of 20.1% over Vandana (Philippines), 2.9% over BRRIdhan 29 (Bangladesh), 9.4% over Hardinath, 30.1% over Tarahara (Nepal) and 18.8% over Shabhagi dhan (India) (Table 7). Grain yield advantage of 1.0% over locally adapted check Shabhagi dhan in India, 13.6% over Vandana in Philippines, 10.9% over BRRIdhan 29 in Bangladesh, 1.4% over Hardinath and 14.1% over Tarahara in Nepal, and 7.0% over TDK11 in Lao PDR was observed for the genotype IR 92521-146-3-3-2 (Vandana/IR 74371-46-1-1//IR 08L183) (Table 7). IR 93835-70-2-2-1 (IR 07L270//Vandana/IR 74371-46-1-1) showed grain yield advantage of 2.9% over Vandana (Philippines), 6.1% over Hardinath, 1.5% over Tarahara (Nepal) and 24.5% over Shabhagi dhan (India) (Table 7). The good yield advantage of the selected genotypes over the presently existing locally adapted varieties, indicates the suitability of these genotypes to be released as variety for cultivation under aerobic conditions. It is important to highlight here that the identified promising genotypes are the progenies of the crosses involving upland adapted breeding varieties/lines-Vandana, IR03A550, drought tolerant breeding lines IR 74371-46-1-1, IR 78877-163-B-1-1, IR 78878-53-2-2-2, and high yielding lowland adapted breeding lines, IR 07L270, IR 08L183, IR 55419-04 in their pedigree, indicating that these are the potential donors in developing varieties suited to aerobic cultivation conditions. The presence of drought QTLs in addition to the QTLs/genes for biotic and abiotic stress tolerance under aerobic conditions indicating the adaptability of genotypes to both drought and aerobic conditions.

**Table 7:** Mean grain yield (Kg ha^−1^) of selected stable genotypes across different ecosystems and QTLs for traits increasing rice adaptation under aerobic situation and resistance/tolerance to biotic stresses.

**Genotype**	**Philippines**	**Bangladesh**		**Nepal**		**India**		**Lao PDR**	**QTLs/gene**
	**IRRI, Los Baños**	**BRRI, Gazipur**	**BRRI, Rajshai**	**NRRP, Hardinath**	**RARS, Tarahara**	**NRRI, Cuttack**	**IRRI SAH, Hyderabad**	**NAFRI, Laos**	
IR 91326-19-2-1-2	5204 (11%)^†^	3661 (14.4%)^†^	–	4830 (11.3%)^†^	3430 (3%)^†^	-	3433 (–)	–	*AG_*9.1*_+GM4+xa4+qDTY_*2.1*_* *+qDTY_*3.1*_+qDTY_*12.1*_*
IR 97041-8-1-1-1	5670 (20.1)^†^	3290 (2.9%)^†^	2991	4750 (9.4%)^†^	4332 (30.1%)^†^	-	4580 (18.8%)^†^	–	*AG_*9.1*_+GM4* *+xa4+Xa21+Pita+qNR_*5.1*_*
IR 92521-146-3-3-2	5328 (13.6%)^†^	3550 (10.9%)^†^	–	4402 (1.4%)^†^	3800 (14.1%)^†^	2815	3891 (1%)^†^	3500 (7.0%)^†^	*AG_*9.1*_+GM4* * +xa4+qDTY_*12.1*_+qDTY_*3.1*_*
IR 93835-70-2-2-1	4830 (2.9%)^†^	2080 (–)	–	4605 (6.1%)^†^	3380 (1.5%)^†^	–	4798 (24.5%)^†^	2889 (–)	*BPH3+xa4+qGY_*6.1*_* *+qDTY_*3.1*_+qRHD_*5.1*_* *+qEVV_*9.1*_*
Vandana^*^	4690	3195	–	3750	2205	1420	2100	–	*AG_*9.1*_+GM4+xa4+qGY_*6.1*_* *+qDTY_*12.1*_*
UPLRi 7^*^	4680	3485	–	3850	3220	1508	1802	–	*AG_*9.1*_+GM4+xa4+qDTY_*3.1*_*
IRRI 132^*^	4110	3305	–	3822	3010	1550	2052	–	*AG_*9.1*_+xa4+qDTY_*2.1*_* *+qDTY_*3.1*_*
BRRIdhan 29^*^	–	3200	–	–	–	–	–	–	*AG_*9.1*_+xa4*
Shabhagi dhan^*^	–	–	–	–	–	2735	3855	–	*AG_*9.1*_+GM4+xa4+NR_*5.1*_* *+qDTY_*2.1*_+qDTY_*3.1*_*
Hardinath^*^	–	–	–	4340	–	–	–	–	–
Tarahara^*^	–	–	–	–	3330	–	–	–	–
TDK1^*^	–	–	–	–	–	–	–	3270	*AG_*9.1*_+Xa4+Pita+Pita2* *+qRHD_*8.1*_+qGY_*6.1*_*
Trial Mean	4992	3796	2870	3780	3127	3927	4423	3420	
LSD (0.05%)	954	396	242	883	386	482	729	721	

* Check variety,

† *percentage grain yield advantage over the respective check in each location; Vandana (Philippines), BRRIdhan 29 (Bangladesh), Hardinath (NRRP Hardinath, Nepal), Tarahara (RARS Tarahara, Nepal), Shabhagi dhan (India), TDK1 (Lao PDR)*.

Root traits such as nodal root and root hair density may impart better nutrient and water uptake under aerobic condition in these genotypes as indicating by the presence of QTLs; *qNR*_5.1_*, qRHD*_5.1_, and *qRHD*_1.1_. In addition to water-labor-energy saving, the identified genotypes with wider adaptability may serve as novel material for aerobic cultivation with yield advantage of at least 0.5–1.0 t ha^−1^ over the existing upland varieties. The release of rice varieties including CR dhan 201, CR dhan 202, and CR dhan 204 for aerobic cultivation in India are successful example of development of aerobic rice at IRRI (Sandhu and Kumar, 2017). The other successful example of aerobic rice varieties includes MAS 946-1 and MAS 26 from University of Agricultural Sciences, Bangalore (Gandhi et al., 2012); Han Dao (HD277, HD 297, HD502) from China Agricultural University (CAU), China.

The genomic regions determining rice adaptation to aerobic situation play an important role in determining the performance of breeding lines under variable aerobic conditions. Interestingly, various marker-assisted introgression studies reported the significant QTL × QTL interactions for drought tolerance related trait in pyramided lines in different backgrounds (Dixit et al., 2012; Kumar et al., 2014; Shamsudin et al., 2016; Vikram et al., 2016; Sandhu et al., 2018). Capturing of such positive interactions for increased performance of QTLs in pyramided lines is necessary in achieving the significant genetic gain. In the present study, *qDTY*_3.1_ either single or in combination with *qDTY*_12.1_ and/or *qDTY*_2.1_, *qGY*_6.1_ provide grain yield stability across different locations under aerobic conditions. A positive interaction of *qDTY*_12.1_ with *qDTY*_2.3_ and *qDTY*_3.2_ (Dixit et al., 2012), *qDTY*_12.1_ with *qDTY*_2.2_ and *qDTY*_3.1_ (Shamsudin et al., 2016), and *qDTY*_7.1_ with *qDTY*_4.1_ and *qDTY*_9.1_ (Sandhu et al., 2018) which significantly increases grain yield under drought have been reported. The genotypes with similar QTL combinations had shown similar yield stability ranking in both the models used in the present study. Introgression of QTLs/gene associated with traits such as anaerobic germination, early uniform emergence, resistance to gall midge and bacterial blight, grain yield under drought and direct seeded conditions may provide grain yield stability under variable growing environments as observed in the present study. There is an urgent need to identify such traits and positive QTLs/gene interactions providing grain yield stability and better nutrient uptake under aerobic conditions.

## Conclusions

This work allowed to identify promising genotypes for targeted rice breeding for adaptation to aerobic systems. The development of breeding lines with 0.5–1.0 t ha^−1^ yield advantage over the existing upland rice varieties and yield stability across different ecosystems and multiple seasons/years would support the cultivation of aerobic rice varieties that can withstand the destabilizing impact of changing climate across Asia. Identification of suitable aerobic cultivars with QTLs/genes providing adaptability and better water-nutrient uptake under shallow rainfed ecosystem shall help extend the domain of aerobic rice cultivation to new areas including irrigated areas with predicted water scarcity and labor shortage problem. The promising genotypes with desirable QTLs/genes may serve as donors in future marker-assisted breeding program. Identification of stable high-yielding genotypes across locations, environments, seasons and years shall help breeders to recommend the identified genotypes for release as varieties to be cultivated by farmers.

## Author Contributions

NS was involved in conducting the experiments at IRRI (Philippines), analysis, data interpretation, and drafting of the manuscript. RY and BC were involved in conducting the experiments at NRRP, Hardinath (Nepal). HP was involved in conducting the experiments at RARS, Tarahara (Nepal). KI was involved in conducting the experiments at BRRI, (Bangladesh). CV was involved in conducting the experiments at Hyderabad (India). AA was involved in conducting the experiments at NRRI, Cuttack (India). PX was involved in conducting the experiments at NAFRI, Lao PDR. KB and MR were involved in conducting the experiment at RRS, Kaul (India). MC, PP, and PM was involved in management of experiments at IRRI (Philippines). KR was involved in data analysis. MC was involved in genotyping. AK conceived the study and was involved in critical revision of the manuscript and final approval of the version to be published.

### Conflict of Interest Statement

The authors declare that the research was conducted in the absence of any commercial or financial relationships that could be construed as a potential conflict of interest.

## References

[B1] Alexandratos N. Bruinsma J. (2012). World Agriculture Towards 2030/2050: The 2012 Revision (No. 12-03 p.), ESA Working paper (Rome: FAO), 4.

[B2] Angaji S. A. Septiningsih E. M. Mackill D. J. Ismail A. M. (2010). QTLs associated with tolerance of flooding during germination in rice (*Oryza sativa* L.). Euphytica 172, 159–168. 10.1007/s10681-009-0014-5

[B3] Atlin G. N. Lafitte H. R. Tao D. Laza M. Amante M. Courtois B. (2006). Developing rice cultivars for high-fertility upland systems in the Asian tropics. Field Crops Res. 97, 43–52. 10.1016/j.fcr.2005.08.014

[B4] Belder P. Bouman B. A. M. Spiertz J. H. J. Peng S. Castaneda A. R. Visperas R. M. (2005). Crop performance, nitrogen and water use in flooded and aerobic rice. Plant Soil 273, 167–182. 10.1007/s11104-004-7401-4

[B5] Bernier J. Kumar A. Ramaiah V. Spaner D. Atlin G. (2007). A large-effect QTL for grain yield under reproductive-stage drought stress in upland rice. Crop Sci. 47, 507–516. 10.2135/cropsci2006.07.0495

[B6] Bouman B. A. M. Lampayan R. M. Tuong T. P. (2007). Water Management in Irrigated Rice: Coping With Water Scarcity. Los Baños: International Rice Research Institute, 54.

[B7] Bouman B. A. M. Wang H. Yang X. Zhao J. F. Wang C. G. (2002). Aerobic rice (Han Dao): a new way of growing rice in water-short areas, in Proceedings of the 12th International Soil Conservation Organization Conference (Beijing), 175–181.

[B8] Bouman B. A. M. Xiaoguang Y. Wang H. Wang Z. Zhao J. Chen B. (2005). Performance of aerobic rice varieties under irrigated conditions in North China. Field Crops Res. 97, 53–61. 10.1016/j.fcr.2005.08.015

[B9] Buresh R. J. Haefele S. M. (2010). Changes in paddy soils under transition to water-saving and diversified cropping systems, in Soil Solutions for a Changing World. 19th World Congress of Soil Science (Brisbane, QLD: Published on DVD).

[B10] Chu Z. Fu B. Yang H. Xu C. Li Z. Sanchez A.. (2006). Targeting *xa13*, a recessive gene for bacterial blight resistance in rice. Theor. Appl. Genet. 112, 455–461. 10.1007/s00122-005-0145-6 16328230

[B11] Connor R. (2015). The United Nations World Water Development Report 2015: Water for a Sustainable World, Vol. 1 UNESCO Publishing.

[B12] Damesa T. M. Möhring J. Worku M. Piepho H. P. (2017). One step at a time: stage-wise analysis of a series of experiments. Agron. J. 109, 845–857. 10.2134/agronj2016.07.0395

[B13] Dixit S. Grondin A. Lee C. R. Henry A. Olds T. M. Kumar A. (2015). Understanding rice adaptation to varying agro-ecosystems: trait interactions and quantitative trait loci. BMC Genet. 16:86. 10.1186/s12863-015-0249-1 26243626 PMC4526302

[B14] Dixit S. Swamy B. P. Vikram P. Ahmed H. U. Cruz M. S. Amante M.. (2012). Fine mapping of QTLs for rice grain yield under drought reveals sub-QTLs conferring a response to variable drought severities. Theor. Appl. Genet. 125, 155–169. 10.1007/s00122-012-1823-9 22361948

[B15] Dou F. Soriano J. Tabien R. E. Chen K. (2016). Soil texture and cultivar effects on rice (*Oryza sativa*, L.) grain yield, yield components and water productivity in three water regimes. PLoS ONE 11:150549. 10.1371/journal.pone.0150549 26978525 PMC4792476

[B16] Eberhart S. A. Russell W. A. (1966). Stability parameters for comparing varieties. Crop Sci. 6, 36–40. 10.2135/cropsci1966.0011183X000600010011x

[B17] Fageria N. (1992). Maximizing Crop Yields. New York, NY: Marcel Dekker; CRC, 274.

[B18] Feng L. Bouman B. A. M. Tuong T. P. Cabangon R. J. Yalong L. Guoan L. (2007). Exploring options to grow rice under water-short conditions in northern China using a modelling approach. I: field experiments and model evaluation. Agric. Water Manage. 88, 1–13. 10.1016/j.agwat.2006.10.006

[B19] Finlay K. W. Wilkinson G. N. (1963). The analysis of adaptation in a plant breeding programme. Austral. J. Agr. Res. 14, 742–754. 10.1071/AR9630742

[B20] Fjellstrom R. Conaway-Bormans C. A. McClung A. M. Marchetti M. A. Shank A. R. Park W. D. (2004). Development of DNA markers suitable for marker assisted selection of three *Pi* genes conferring resistance to multiple *Pyricularia grisea* pathotypes. Crop Sci. 44, 1790–1798. 10.2135/cropsci2004.1790

[B21] Gandhi R. V. Rudresh N. S. Shivamurthy M. Hittalmani S. (2012). Performance and adoption of new aerobic rice variety MAS 946-1 (Sharada) in southern Karnataka. Kar. J. Agric. Sci. 25, 5–8.

[B22] Gao X. P. Zou C. Q. Fan X. Y. Zhang F. S. Hoffland E. (2006). From flooded to aerobic conditions in rice cultivation: consequences for zinc uptake. Plant Soil 280, 41–47. 10.1007/s11104-004-7652-0

[B23] Gauch H. G. Jr. (1988). Model selection and validation for yield trials with interaction. Biometrics 44, 705–715. 10.2307/2531585

[B24] Gauch H. G. Jr. (1992). Statistical Analysis of Regional Yield Trials: AMMI Analysis of Factorial Designs. Washington, DC: Elsevier Science Publishers.

[B25] George T. Magabanua R. Garrity D. P. Tubana B. S. Quiton J. (2002). Rapid yield loss of rice cropped successively in aerobic soil. Agron. J. 84, 981–989. 10.2134/agronj2002.0981

[B26] Jairin J. Phengrat K. Teangdeerith S. Vanavichit A. Toojinda T. (2007). Mapping of a broad-spectrum brown planthopper resistance gene, *Bph3*, on rice chromosome 6. Mol. Breed. 19, 35–44. 10.1007/s11032-006-9040-3

[B27] Khowaja F. S. Norton G. J. Courtois B. Price A. H. (2009). Improved resolution in the position of drought-related QTLs in a single mapping population of rice by meta-analysis. BMC Genomics 10:276. 10.1186/1471-2164-10-276 19545420 PMC2708188

[B28] Koide Y. Ebron L. A. Kato H. Tsunematsu H. Telebanco-Yanoria M. J. Kobayashi N. (2011). A set of near-isogenic lines for blast resistance genes with an Indica-type rainfed lowland elite rice (*Oryza sativa* L.) genetic background. Field Crops Res. 123, 19–27. 10.1016/j.fcr.2011.04.005

[B29] Kreye C. Bouman B. A. M. Castañeda A. R. Lampayan R. M. Faronilo J. E. Lactaoen A. (2009). Possible causes of yield failure in tropical aerobic rice. Field Crops Res. 111, 197–206. 10.1016/j.fcr.2008.12.007

[B30] Kumar A. Dixit S. Ram T. Yadaw R. B. Mishra K. K. Mandal N. P. (2014). Breeding high-yielding drought-tolerant rice: genetic variations and conventional and molecular approaches. J. Exp. Bot. 65, 6265–6278. 10.1093/jxb/eru363 25205576 PMC4223988

[B31] Kumar V. Ladha J. K. (2011). Direct seeding of rice: recent developments and future research needs. Adv. Agron. 111, 297–413. 10.1016/B978-0-12-387689-8.00001-1

[B32] Kumar V. Ladha J. K. Gathala M. K. (2009). Direct drill-seeded rice: a need of the day, in Annual Meeting of Agronomy Society of America (Pittsburgh, PA). Available online at: http://a-c-s.confex.com/crops/2009am/webprogram/Paper53386.html

[B33] Li Y. H. (2001). Research and practice of water-saving irrigation for rice in China, in Proceedings of an International Workshop, Water-Saving Irrigation for Rice, 23-25 March 2001, eds Barker R. Li Y. Tuong T. P. (Wuhan; Colombo: International Water Management Institute), 135–144.

[B34] Malosetti M. Ribaut J. M. van Eeuwijk F. A. (2013). The statistical analysis of multi-environment data: modeling genotype-by-environment interaction and its genetic basis. Front. Physiol. 4:44. 10.3389/fphys.2013.00044 23487515 PMC3594989

[B35] Monaco F. Sali G. Ben Hassen M. Facchi A. Romani M. Valè G. (2016). Water management options for rice cultivation in a temperate area: a multi-objective model to explore economic and water saving results. Water 8, 336–355. 10.3390/w8080336

[B36] Nair S. Kumar A. Srivastava M. N. Mohan M. (1996). PCR-based DNA markers linked to a gall midge resistance gene, Gm4t, has potential for marker-aided selection in rice. Theor. Appl. Genet. 92, 660–665. 10.1007/BF00226086 24166388

[B37] Nie L. Peng S. Bouman B. A. M. Huang J. Cui K. Visperas R. M. (2008). Alleviating soil sickness caused by aerobic monocropping: responses of aerobic rice to nutrient supply. Field Crops Res. 107, 129–136. 10.1016/j.fcr.2008.01.006

[B38] Nie L. Peng S. Chen M. Shah F. Huang J. Cui K. (2012). Aerobic rice for water-saving in agriculture: a review. Agron. Sustain. Dev. 32, 411–418. 10.1007/s13593-011-0055-8

[B39] Peng S. Bouman B. A. M. Visperas R. M. Castañeda A. R. Nie L. Park H. K. (2006). Comparison between aerobic and flooded rice in the tropics: agronomic performance in an eight-season experiment. Field Crops Res. 96, 252–259. 10.1016/j.fcr.2005.07.007

[B40] Perumalsamy S. Bharani M. Sudha M. Nagarajan P. Arul L. Saraswathi R. (2010). Functional marker-assisted selection for bacterial leaf blight resistance genes in rice (*Oryza sativa* L.). Plant Breed. 129, 400–406. 10.1111/j.1439-0523.2009.01705.x

[B41] Piepho H. P. (1998). Methods for comparing the yield stability of cropping systems - a review, J. Agron. Crop Sci. 180, 193–213.

[B42] Piepho H. P. (1999). Stability analysis using the SAS system. Agron. J. 9, 154–160. 10.2134/agronj1999.00021962009100010024x

[B43] Pinheiro B. D. S. de Castro E. D. M. Guimaraes C. M. (2006). Sustainability and profitability of aerobic rice production in Brazil. Field Crops Res. 97, 34–42. 10.1016/j.fcr.2005.08.013

[B44] Qu S. Liu G. Zhou B. Bellizzi M. Zeng L. Dai L.. (2006). The broad-spectrum blast resistance gene Pi9 encodes a nucleotide-binding site–leucine-rich repeat protein and is a member of a multigene family in rice. Genetics 172, 1901–1914. 10.1534/genetics.105.044891 16387888 PMC1456263

[B45] Raman A. Ladha J. K. Kumar V. Sharma S. Piepho H. P. (2011). Stability analysis of farmer participatory trials for conservation agriculture using mixed models. Field Crops Res. 121, 450–459. 10.1016/j.fcr.2011.02.001

[B46] Rashid M. H. Alam M. M. Khan M. A. H. Ladha J. K. (2009). Productivity and resource use of direct-(drum)-seeded and transplanted rice in puddled soils in rice-rice and rice-wheat ecosystem. Field Crops Res. 113, 274–281. 10.1016/j.fcr.2009.06.004

[B47] Rasul G. (2016). Managing the food, water, and energy nexus for achieving the Sustainable Development Goals in South Asia. Environ. Dev. 18, 14–25. 10.1016/j.envdev.2015.12.001

[B48] Sama A. Himabindu K. Bhaskar S. N. Sundaram R. M. Viraktamath B. C. Bentur J. (2012). Mapping and marker-assisted breeding of a gene allelic to the major Asian rice gall midge resistance gene Gm8. Euphytica 187, 393–400. 10.1007/s10681-012-0724-y

[B49] Sandhu N. Dixit S. Swamy B. P. M. Vikram P. Venkateshwarlu C. Catolos M.. (2018). Positive interactions of major-effect QTLs with genetic background that enhances rice yield under drought. Sci. Rep. 8:1626. 10.1038/s41598-018-20116-7 29374240 PMC5786057

[B50] Sandhu N. Kumar A. (2017). Bridging the rice yield gaps under drought: QTLs, genes, and their use in breeding programs. Agronomy 7:27 10.3390/agronomy7020027

[B51] Sandhu N. Torres R. O. Cruz M. T. S. Maturan P. C. Jain R. Kumar A.. (2015). Traits and QTLs for development of dry direct-seeded rainfed rice varieties. J. Exp. Bot. 66, 225–244. 10.1093/jxb/eru413 25336682 PMC4265160

[B52] Shamsudin N. A. Swamy B. M. Ratnam W. Cruz M. T. S. Raman A. Kumar A. (2016). Marker assisted pyramiding of drought yield QTLs into a popular Malaysian rice cultivar, MR219. BMC Genet. 17:30. 10.1186/s12863-016-0334-0 26818269 PMC4729146

[B53] Shikari A. B. Khanna A. Krishnan S. G. Singh U. D. Rathour R. Tonapi V. (2013). Molecular analysis and phenotypic validation of blast resistance genes *Pita* and *Pita2* in landraces of rice (*Oryza sativa* L.). Indian J. Genet. 73, 131–141. 10.5958/j.0975-6906.73.2.020

[B54] Shukla G. K. (1972). Some statistical aspects of partitioning genotype environmental components of variability. Heredity 29, 237–245. 10.1038/hdy.1972.87 4507945

[B55] Smith A. B. Cullis B. R. Gilmour A. (2001). The analysis of crop variety evaluation data in Australia. Aust. N. Zeal. J. Stat. 43, 129–145. 10.1111/1467-842X.00163

[B56] Smith A. B. Cullis B. R. Thompson R. (2005). The analysis of crop cultivar breeding and evaluation trials: an overview of current mixed model approaches. J. Agric. Sci. 143, 449–462. 10.1017/S0021859605005587

[B57] Song W. Y. Pi L. Y. Wang G. L. Gardner J. Holsten T. Ronald P. C. (1997). Evolution of the rice *Xa21* disease resistance gene family. Plant Cell 9, 1279–1287. 10.1105/tpc.9.8.1279 9286106 PMC156997

[B58] Stoop W. Uphoff N. Kassam A. (2002). A review of agricultural research issues raised by the system of rice intensification (SRI) from Madagascar: opportunities for improving farming systems for resource-poor farmers. Agric. Syst. 71, 249–274. 10.1016/S0308-521X(01)00070-1

[B59] Sun L. Su C. Wang C. Zhai H. Wan J. (2005). Mapping of a major resistance gene to the brown planthopper in the rice cultivar Rathu Heenati. Breed. Sci. 55, 391–396. 10.1270/jsbbs.55.391

[B60] Swamy P. Panchbhai A. N. Dodiya P. Naik V. Panchbhai S. D. Zehr U. B. (2006). Evaluation of bacterial blight resistance in rice lines carrying multiple resistance genes and *Xa21* transgenic lines. Curr. Sci. 90, 818–824.

[B61] Tabbal D. F. Bouman B. A. M. Bhuiyan S. I. Sibayan E. B. Sattar M. A. (2002). On-farm strategies for reducing water input in irrigated rice: case studies in the Philippines. Agric. Water Manag. 56, 93–112. 10.1016/S0378-3774(02)00007-0

[B62] Tuong T. P. Bouman B. A. M. Mortimer M. (2005). More rice, less water: integrated approaches for increasing water productivity in irrigated rice-based systems in Asia. Plant Prod. Sci. 8, 231–241. 10.1626/pps.8.231

[B63] Ullah I. Jamil S. Iqbal M. Z. Shaheen H. L. Hasni S. M. Jabeen S.. (2012). Detection of bacterial blight resistance genes in basmati rice landraces. Genet. Mol. Res. 11, 1960–1966. 10.4238/2012.July.20.1 22869552

[B64] USGS (2016). USGSFact Sheet. Available online at: http://water.usgs.gov/edu/gwdepletion.html

[B65] Venuprasad R. Dalid C. O. Del Valle M. Zhao D. Espiritu M. Cruz M. T. S.. (2009). Identification and characterization of large-effect quantitative trait loci for grain yield under lowland drought stress in rice using bulk-segregant analysis. Theor. Appl. Genet. 120, 177–190. 10.1007/s00122-009-1168-1 19841886

[B66] Venuprasad R Bool M. E. Quiatchon L. Sta Cruz M. T. Amante M. Atlin G. N. (2012). A large effect QTL for rice grain yield under upland drought stress on chromosome 1. Mol. Breed. 30, 535–547. 10.1007/s11032-011-9642-2

[B67] Vikram P. Swamy B. M. Dixit S. Ahmed H. U. Cruz M. T. S. Singh A. K.. (2011). *qDTY*_1.1_ a major QTL for rice grain yield under reproductive-stage drought stress with a consistent effect in multiple elite genetic backgrounds. BMC Genet. 12:89. 10.1186/1471-2156-12-89 22008150 PMC3234187

[B68] Vikram P. Swamy B. M. Dixit S. Trinidad J. Cruz M. T. S. Maturan P. C.. (2016). Linkages and interactions analysis of major effect drought grain yield QTLs in rice. PloS ONE 11:e0151532. 10.1371/journal.pone.0151532 27018583 PMC4809569

[B69] Wang H. Boumam B. A. M. Zhao D. Wang C. Moya P. F. (2002). Aerobic rice in northern China: opportunities and challenges, in Proceedings of the International Workshop on Water-Wise Rice Production, Water-Wise Rice Production, 8-11 April, eds Bouman B. A. M. Hengsdijk H. Hardy B. Bindraban P. S. Tuong T. P. Ladha J. K. (Los Baños: International Rice Research Institute), 143–154.

[B70] Weller S. Janz B. Jörg L. Kraus D. Racela H. S. Wassmann R.. (2016). Greenhouse gas emissions and global warming potential of traditional and diversified tropical rice rotation systems. Glob. Change Biol. 22, 432–448. 10.1111/gcb.13099 26386203

[B71] WWAP (2016). The United Nations World Water Development Report 2016: Water and Jobs. United Nations World Water Assessment Programme Paris: UNESCO.

[B72] Xiaoguang Y. Bouman B. A. M. Huaqi W. Zhimin W. Junfang Z. Bin C. (2005). Performance of temperate aerobic rice under different regimes in North China. Agric. Water Manage. 74, 107–122. 10.1016/j.agwat.2004.11.008

[B73] Yamano T. Baruah S. Sharma R. Kumar A. (2013). Factors Affecting the Adoption of Direct-Seeded Rice in the Northeastern Indo-Gangetic Plain. Report. Cereal Systems Initiative for South Asia.

[B74] Zhao D. L. Atlin G. N. Amante M. Sta Cruz M. T. Kumar A. (2010). Developing aerobic rice cultivars for water-short irrigated and drought-prone rainfed areas in the tropics. Crop Sci. 50, 2268–2276. 10.2135/cropsci2010.10.0028

