# Supplementary material for: Evaluating the Performance of Rice Genotypes for Improving Yield and Adaptability Under Direct Seeded Aerobic Cultivation Conditions
Source: Front Plant Sci. 2019 Feb 15;10:159. doi: 10.3389/fpls.2019.00159 (PMC6384261; doi:10.3389/fpls.2019.00159)
Supplement: Supplementary file 1 [file Table_1.DOCX]

| **QTLs/gene** | **Markers** | **References** |
| --- | --- | --- |
| *qAG_9.1_* | DFR_F2, DFR_R2, DFR_LB2 | Angaji et al., 2010 |
| *GM4* | gene specific markers (GM4_LRR-del_F, GM4_LRR-del_R) | Nair et al.,1996; Sama et al., 2012 |
| *xa4* | gene specific markers (xa4_F, xa4_R) | Ullah et al., 2012; Perumalsamy et al., 2010; Chu et al., 2006; Swamy et al., 2004, Song et al., 1997 |
| *xa5* | gene specific markers (xa5_F2_Sus, xa5_F2_Res, xa5_R2) |  |
| *XA13* | gene specific markers (xa13F_130-147, xa13R_1678-1662) |  |
| *XA21* | gene specific markers (M769, M770) |  |
| *Pi9* | gene specific markers (M492, M493) | Qu et al., 2006; Koide et al., 2011; Fjellstrom et al., 2004; Shikari et al., 2013, IRRI |
| *Pita2* | gene specific markers (M535, M536) |  |
| *BPH3* | RM586, RM589, RM7639, RM19311, RM190 | Jairin et al., 2007; Sun et al. 2005 |
| *BPH17* | RM8213, RM6487, RM16430, RM16431, RM16556, RM16567 |  |
| *qGY_1.1_* | RM11943, RM6333, RM431, RM12147, RM5310, RM122281, RM12092, RM12289, RM12276 | Sandhu et al., 2015 |
| *qGY_6.1_* | RM20493, RM20535, RM20632, RM20633 |  |
| *qGY_10.1_* | RM25457, RM25745, RM1108, RM25895 |  |
| *qNR_4.1_* | RM5414, RM16424, RM16428, RM8213, RM6487, RM1305, RM16556, RM16686, RM16672 |  |
| *qRHD_5.1_* | RM18166, RM18149, RM18173, RM18354, RM18360 |  |
| *qRHD_8.1_* | RM22306, RM1376 |  |
| *qRHD_1.1_* | RM5989, RM8098, RM6784, RM259, RM1032, RM10701 |  |
| *qEVV_9.1_* | RM24351 |  |
| *qNR_5.1_* | RM17885, RM3345, RM5796 |  |
| *qEMM_1.1_* | RM10012, RM10043, RM10076, RM6887, RM495 | Dixit et al., 2015 |
| *qEMM_11.1_* | RM26076, RM26092, RM26279, RM26321 |  |
| *qDTY_1.1_* | RM431, RM11943, RM12023, RM12146, RM12233 | Vikram et al., 2011 |
| *qDTY_2.1_* | RM324, RM3549, RM12868, RM5791, RM12987, RM12995 | Venuprasad et al., 2009 |
| *qDTY_3.1_* | RM520, RM416, RM16030 |  |

**Supplementary Table 1** Details on markers used to identify QTLs/gene present in the selected promising lines
